# Determinants of medication adherence in patients with diabetes, hypertension, and hyperlipidemia

**DOI:** 10.1007/s42000-025-00631-9

**Published:** 2025-02-20

**Authors:** Athanasios Chantzaras, John Yfantopoulos

**Affiliations:** MBA Health Economics & Management School of Economics and Political Sciences, National and Kapodistrian University of Athens, 6 Themistokleous Street, 10678, Athens, Greece

**Keywords:** Medication adherence, Diabetes, Hypertension, Hyperlipidemia, Greece

## Abstract

**Objectives:**

To investigate medication non-adherence and its determinants in diabetes, hypertension, and hyperlipidemia.

**Methods:**

In a multicenter, cross-sectional, non-interventional study, 518 diabetic, 721 hypertensive, and 463 hyperlipidemic patients were recruited, using consecutive sampling, in Greece during the COVID-19 pandemic. Medication adherence was measured with the Adherence to Refills and Medications Scale (ARMS). Multiple linear regressions with robust standard errors investigated the predictors of the ARMS summary score.

**Results:**

Perfect adherence was estimated at 16%, 12%, and 11%, and low adherence at 38.8%, 61.3%, and 66.7% in diabetes, hypertension, and hyperlipidemia, respectively. The factors that significantly increased the likelihood of non-adherence were the following: (a) lower age, female gender, no public health insurance, high perceived threat of illness, low satisfaction with physician consultations, shorter consultations, bad general health, fewer comorbidities, and type 2 diabetes; (b) male gender, not being married, low education, no public insurance, smoking, frequent drinking, shorter consultations, self-perceived inadequacy of knowledge, negative views of medication, presence of comorbidities, fewer medicines being used, and high blood pressure in hypertension; and (c) lower age, not being employed, smoking, frequent drinking, no public insurance, low satisfaction with consultations, negative views of medication, taking 3–4 medicines, high LDL, and low HDL and triglyceride levels in hyperlipidemia. Different curvilinear associations of adherence with BMI and exercise were also found.

**Conclusion:**

Medication non-adherence is very common in diabetes, hypertension, and hyperlipidemia. Strategies to improve adherence should consider the different determinants of non-adherence among patient groups.

## Introduction

Over the past few decades, the adverse impact of chronic diseases on populations’ health and quality of life has been growing. An ever increasing number of people are today living with chronic diseases for numerous reasons, such as *ageing of the population*,* changing lifestyle factors*,* medical advances*,* improved living conditions*,* and better prevention and infectious disease management* excessive alcohol and tobacco use, poor nutrition, lack of physical activity/sedentary lifestyle, and longer lifespans—which latter, while excellent in itself, increases the prevalence of age-related illnesses and chronic conditions. [1]Today, chronic conditions are the leading cause of morbidity and mortality around the world, being responsible for more than 36 million deaths annually [2]. Furthermore, chronic diseases not only increase the risk of severe morbidity and mortality but are also associated with various other negative consequences at the individual, healthcare system, and societal levels [3].

Medication non-adherence, which refers to the failure to take medicines as prescribed, may consist of non-initiation, suboptimal implementation, or early discontinuation of treatment [4]. Medication non-adherence is a very common issue among patients suffering from chronic conditions, the rate having been estimated at approximately 50% on average in developed countries [5]. Poor adherence leads to suboptimal health outcomes and increased morbidity and mortality as well as to considerable additional healthcare costs, being instrumental in aggravating the already low efficiency of health systems [5].

Diabetes, hypertension, and hyperlipidemia are among the most prevalent chronic conditions worldwide. Despite treatment advances, diabetes continues to impose a substantial clinical and economic burden on patients and the society as a whole. Nevertheless, even though good medication compliance can improve glycemic control in this disease, several studies have reported suboptimal adherence rates among diabetes patients [6–9]. Moreover, while blood pressure control is crucial in reducing cardiovascular risk, the majority of hypertensive patients do not achieve and maintain well-controlled blood pressure. In this regard, poor adherence to blood-pressure-lowering medications is widely acknowledged as the main explanation for treatment failure in hypertension [10]. Furthermore, hyperlipidemia is a principal risk factor for the development of cardiovascular conditions and is the cause of more than 2.6 million deaths annually [11]. Again, despite the benefits of antihyperlipidemic agents, adherence to statin therapy remains a challenge worldwide [12].

Treatment adherence is a complex process that is influenced by numerous factors related to the healthcare system, condition, therapy, patient, and socioeconomic background [5]. Better understanding of non-adherence and its predictors is crucial, and especially nowadays owing to the increasing number of self-administered medicines. The primary aim of this research work was to investigate the prevalence of medication non-adherence and its determinants in patients with diabetes, hypertension, and hyperlipidemia in Greece.

## Methods

### Study design and participants

This multicenter, cross-sectional, non-interventional study was conducted between March 2020 and March 2021 in Greece. Patients were recruited, using a consecutive sampling method, from the outpatient departments of different general public and private hospitals, community pharmacies, and private practices, and from patient groups and medical societies across the country. No specific treatment protocol or visit program was applied and participants were treated according to routine clinical practice.

Subjects eligible for inclusion in this study were 18 years or older, with a confirmed physician diagnosis of diabetes, hypertension, or hyperlipidemia, who had been receiving treatment for their condition for at least 6 months. Pregnant women, patients not mentally capable of comprehending and answering the survey questionnaire or not able or willing to provide written consent, and those with an acute life-threatening condition were excluded.

Data collection was completely anonymous and all participants provided written informed consent. The study was conducted according to the principles of the Declaration of Helsinki. Ethical approval for conducting this study was granted by the relevant institutions.

### Data collection

The survey instrument was designed to address the specific objectives of this study based on a comprehensive review of the literature. The instrument was pilot-tested before the launch of the main fieldwork to improve it in terms of feasibility, readability, and comprehensibility. Data collection was conducted by trained field investigators via face-to-face interviews with the study participants. Information was collected on sociodemographics, health behaviors, the patients’ beliefs about medicines and their disease, medication adherence, and clinical characteristics.

Medication adherence was measured with the Adherence to Refills and Medications Scale (ARMS) [13]. ARMS is a validated 12-item instrument that consists of two subscales, as follows: (a) adherence to taking medications (eight items) and (b) adherence to refilling prescriptions (four items). Each item is rated by participants on a 4-point Likert frequency scale, as follows: 1 = none of the time, 2 = some of the time, 3 = most of the time, and 4 = all of the time. Item scores are summed to produce an overall medication adherence score ranging between 12 and 48 (one item is reverse coded), with lower scores indicating better medication adherence. A major advantage of the ARMS tool is its suitability for use among chronically ill patients, especially those with low literacy skills [13]. The minimum ARMS summary score of 12 was used to indicate perfect medication adherence [14]. The median of the summary score (= 16) classified participants into the low and high adherent groups. The same cut-off has also been applied in previous studies [15, 16]. The ARMS summary scale was found to have high internal consistency in all patient groups (standardized Cronbach’s alpha: 0.781 for diabetes, 0.869 for hypertension, and 0.897 for hyperlipidemia).

The Beliefs about Medicines Questionnaire (ΒΜQ) [17] evaluates the cognitive representations of patients about their medication and has previously been used in several studies in Greece [18, 19]. It is composed of two parts, as follows: (a) the BMQ-specific, which assesses patients’ beliefs about their prescribed medicines (antidiabetic, antihypertensive, and antihyperlipidemic) and (b) the BMQ-general, which examines patients’ beliefs about medicines in general. The BMQ-specific, which was used in this study, is a 10-item questionnaire which incorporates the following two subscales: (a) the Specific-Necessity subscale, which contains five items assessing patients’ beliefs on the necessity of the prescribed medication, and (b) the Specific-Concern subscale, which consists of five items assessing patients’ concerns about potential adverse consequences of taking their medication. Participants indicate their level of agreement with each statement (item) on a 5-point Likert scale, from 1 = strongly disagree to 5 = strongly agree. Scores obtained for the individual items of the BMQ-Specific were summed to give a score ranging from 5 to 25 for each subscale. Higher score values signify stronger beliefs in the concepts represented by each scale. The Necessity-Concerns subscales differential (NCD) was calculated by subtracting the Concern subscale score from the Necessity subscale score for each individual, leading to a value between − 20 and 20. A positive value indicates that the patient had fewer concerns and stronger ideas of need for their prescribed medicines. In essence, the perceived benefits (necessity) of the treatment are weighted against its perceived *risks and it is a method of a cost-benefit decision patients are making with their medications* / risks, this being a method evaluating to what extent patients are making a cost-benefit decision regarding their medications [20]. The standardized Cronbach’s alpha values were again rated ‘acceptable to high’ for both dimensions and for all patient groups (necessity subscale: 0.835 for diabetes, 0.765 for hypertension, and 0.741 for hyperlipidemia; concerns subscale: 0.788 for diabetes, 0.754 for hypertension, and 0.689 for hyperlipidemia; and for the whole specific part: 0.788 for diabetes, 0.754 for hypertension, and 0.689 for hyperlipidemia).

The Brief Illness Perception Questionnaire (BIPQ), which is the short version of the Illness Perception Questionnaire-Revised (IPQ-R), evaluates the cognitive and emotional beliefs of patients about the disease [21, 22]. This questionnaire has also previously been used in prior research in Greece [19, 23–25]. The BIPQ is a 9-item instrument. Five items assess the cognitive representations of disease (consequences, timeline of disease, personal and treatment control, and identity), two items evaluate emotional representations (concern and emotional response), and one item assesses comprehensibility. The BIPQ instrument also has an open-ended response question which was not used in this study. Each item is rated on a continuous scale from 0 to 10, and the total score ranges from 0 to 80 (three questions are reverse coded). A higher score reflects a more threatening view of the illness. A BIPQ summary score equal to or above the median (= 40) was considered to represent a belief that the condition is associated with a high threat. The standardized Cronbach’s alpha values were lower than the other standardized instruments (0.613 for diabetes, 0.684 for hypertension, and 0.706 for hyperlipidemia).

### Statistical analysis

Descriptive statistics were used to summarize patient characteristics and medication adherence. Univariate group differences with respect to the ARMS summary score were investigated with the Mann-Whitney U and the Kruskal-Wallis tests for independent variables with two and three or more groups, respectively. Multiple linear regressions with robust standard errors were carried out to identify the independent predictors of medication adherence for each patient group. Multicollinearity was explored using the variance inflation factor. Type of diabetic medication was excluded from the model due to high collinearity with type of diabetes.

The level of significance was set at α = 0.05. Statistical analysis was performed using STATA^®^ v.17.

## Results

A total of 518 diabetic, 721 hypertensive, and 463 hyperlipidemic patients participated in this study (overall response rate: 88%). Patient sociodemographics and clinical characteristics are summarized in Table 1. The majority of the participants with diabetes were females (63.9%), while most of the subjects with hypertension and hyperlipidemia were males (57% and 64.6%, respectively). Diabetic patients were on average younger (mean age: 47.4 years) than the hypertensive (67.9 years) and hyperlipidemic (68.7 years) patients. As the cohorts were older *for*? / in the hypertension and hyperlipidemia groups, the share of participants with high education level was much lower (20% and 37.8%, respectively) compared with the diabetes group (53.3%). Most of the patients were highly satisfied with their physician consultation/interaction; however, about one-third of the sample did not consider themselves well-informed about their medicines. Notably, 90.5% of diabetic patients believed that the benefits of taking their medications outweigh their associated risks, whereas the respective estimates were much lower in hypertension (77.5%) and hyperlipidemia (24%). Furthermore, 75.1% of participants with diabetes held high threatening illness perceptions compared with the 42.6% and 25.1% of patients with hypertension and hyperlipidemia, respectively.

**Table 1:** Patients’ sociodemographics and clinical characteristics

Factors	Diabetes		Hypertension		Hyperlipidemia	
	*n*	%	*n*	%	*n*	%
**Sex**						
Male	187	36.1	411	57.0	299	64.6
Female	331	63.9	310	43.0	164	35.4
**Age**						
Mean (SD)	47.36 (41.78)		67.88 (11.60)		68.74 (14.55)	
≤ 49 years	320	61.8	50	6.9	61	13.2
50–59 years	122	23.6	126	17.5	47	10.2
60–69 years	43	8.3	186	25.8	109	23.5
≥ 70 years	33	6.4	359	49.8	246	53.1
**Married**						
No	232	44.8	240	33.3	152	32.8
Yes	286	55.2	481	66.7	311	67.2
**Employed**						
Yes	284	54.8	272	37.7	173	37.4
No	234	45.2	449	62.3	290	62.6
**Education level**						
Low education	242	46.7	577	80.0	288	62.2
High education	276	53.3	144	20.0	175	37.8
**Health insurance status**						
Public	401	77.4	568	78.8	333	71.9
Public & private	66	12.7	121	16.8	107	23.1
No health insurance or only private	51	9.8	32	4.4	23	5.0
**BMI categories**						
Underweight or normal weight	191	36.9	245	34.0	156	33.7
Overweight	164	31.7	365	50.6	173	37.4
Obese	163	31.5	111	15.4	134	28.9
**Smoking**						
No	310	59.8	502	69.6	283	61.1
Yes	208	40.2	219	30.4	180	38.9
**Drinking alcohol frequency**						
2–4 times per month or less	436	84.2	606	84.0	407	87.9
At least 2–3 times weekly	82	15.8	115	16.0	56	12.1
**Exercise per week**						
0 days	101	19.5	363	50.3	205	44.3
1–2 days	182	35.1	212	29.4	118	25.5
3 + days	235	45.4	146	20.2	140	30.2
**Patient satisfaction with consultation**						
Low satisfaction	49	9.5	71	9.8	108	23.3
High satisfaction	469	90.5	650	90.2	355	76.7
**Consultation length**						
≤ 10 min	25	4.8	107	14.8	82	17.7
11–20 min	175	33.8	306	42.4	153	33.0
≥ 21 min	318	61.4	308	42.7	228	49.2
**Self-perceived knowledge adequacy about the medication**						
Yes	352	68.0	456	63.2	295	63.7
No	166	32.0	265	36.8	168	36.3
**BMQ NCD**						
Negative cost-benefit	30	5.8	162	22.5	111	24.0
Positive cost-benefit	488	94.2	559	77.5	352	76.0
**BIPQ Level of illness threat**						
Low threat	129	24.9	414	57.4	347	74.9
High threat	389	75.1	307	42.6	116	25.1
**Good or very good general health**						
No	187	36.1	376	52.1	293	63.3
Yes	331	63.9	345	47.9	170	36.7
**Number of comorbidities**						
0	205	39.6	208	28.8	100	21.6
1	117	22.6	277	38.4	163	35.2
≥ 2	196	37.8	236	32.7	200	43.2
**Time taking medication for main disease**						
0–4 years	50	9.7	113	15.7	129	27.9
5–9 years	94	18.1	162	22.5	136	29.4
10–19 years	163	31.5	272	37.7	139	30.0
20 + years	211	40.7	174	24.1	59	12.7
**Total number of medications (for all conditions)**						
1–2	225	43.4	279	38.7	147	31.7
3–4	180	34.7	247	34.3	169	36.5
5+	113	21.8	195	27.0	147	31.7
Type of diabetes						
Type1	342	66.0				
Type2	176	34.0				
**Type of antidiabetic medication**						
Only oral medication	176	34.0				
Insulin with or without oral medication	342	66.0				
**HbA1c**						
Less than 7%	221	42.7				
Greater than or equal to 7%	297	57.3				
**Blood pressure**						
Greater than 130/80 mm Hg			390	54.1		
Less than or equal to 130/80 mm Hg			331	45.9		
**LDL**						
Greater than or equal to 100 mg/dL)					285	61.6
Less than 100 mg/dL					160	34.6
**HDL**						
Less than or equal to 40 mg/dL for male or less than or equal to 50 mg/dL for female					160	34.6
Greater than 40 mg/dL for male or greater than 50 mg/dL for female					285	61.6
**Triglyceride**						
Greater than or equal to 150 mg/dL					301	65.0
Less than 150 mg/dL					144	31.1

Note: Values represent n and %, unless stated otherwise. Abbreviation: n, number of patients; SD, standard deviation; BIPQ, Brief Illness Perception Questionnaire; BMI, body mass index; BMQ NCD, Beliefs about Medicines Questionnaire necessity-concerns scales differential; HbA1C, hemoglobin A1C; HDL, high-density lipoprotein; LDL, low-densitylipoprotein

Tables 2, 3 and 4 summarize the results of the responses to the ARMS questionnaire for the three patient groups. For diabetes, the most adherent behavior is observed for the elements of economic barriers to refilling the prescription, missing a dose when feeling sick, skipping a dose just before visiting the doctor, and deciding not to take the medicines. For hypertension, patients were more adherent with respect to the items of missing doses when feeling sick, economic barriers to refilling the prescription, running out of medicines, changing doses according to personal needs, forgetting to fill prescriptions, and deciding not to take the medication. For hyperlipidemia, the most adherent behavior was identified *for the items of* / with the items regarding the following: deciding not to take medicines, economic barriers to refilling, changing doses according to personal needs, and missing doses when feeling sick. On the other hand, poor adherence to timely prescription refill was by far the most frequently reported problem in all three conditions. Overall, suboptimal medication adherence was observed in all patient groups, with only 11% of hyperlipidemic patients being perfectly adherent, followed by 12.2% of hypertensive and 15.8% of diabetic patients. Moreover, the prevalence of poor adherence was 38.8% in diabetes, 61.3% in hypertension, and 66.7% in hyperlipidemia. Finally, the mean ARMS summary score was 15.6 (± 3.5), 18.6 (± 5.4), and 19.1 (± 5.2) in the three patient groups.

**Table 2:** ARMS questionnaire responses (%) in diabetes

Items	None of the time	Some of the time	Most of the time	All the time
How often do you forget to take your medicine?	73.9	22.2	3.5	0.4
How often do you decide not to take your medicine?	89.0	9.5	0.8	0.8
How often do you forget to get prescriptions filled?	84.7	12.9	1.5	0.8
How often do you run out of medicine?	64.5	22.2	6.9	6.4
How often do you skip a dose of your medicine before you go to the doctor?	90.7	8.1	1.0	0.2
How often do you miss taking you medicine when you feel better?	87.3	8.1	3.7	1.0
How often do you miss taking your medicine when you feel sick?	91.3	6.8	1.2	0.8
How often do you miss taking your medicine when you are careless?	71.0	24.1	4.6	0.2
How often do you change the dose of your medicines to suit your needs (like when you take more or less pill than you’re supposed to)?	53.7	35.1	6.8	4.4
How often do you forget to take your medicine when you are supposed to take it more than once a day?	81.5	16.6	1.7	0.2
How often do you put off refilling your medicines because they cost too much money?	91.9	6.4	1.7	0.0
How often do you plan ahead and refill your medicines before they run out?	8.9	9.5	28.4	53.3
**Perfect adherence (summary score = 12)**				
Non-adherent	84.2			
Perfectly adherent	15.8			
**Level of adherence**				
Low adherence	38.8			
High adherence	61.2			
**Adherence to taking medications correctly subscale**, mean (SD)	10.01 (2.63)			
**Adherence to refilling medications on schedule subscale**, mean (SD)	5.57 (1.63)			
**ARMS summary score**, mean (SD)	15.58 (3.48)			

Note: Values represent n and %, unless stated otherwise. Abbreviations: n, number of patients; SD, standard deviation; ARMS, Adherence to Refills and Medications Scale

**Table 3:** ARMS questionnaire responses (%) in hypertension

Items	None of the time	Some of the time	Most of the time	All the time
How often do you forget to take your medicine?	52.8	37.4	9.7	0.0
How often do you decide not to take your medicine?	59.6	31.5	8.7	0.1
How often do you forget to get prescriptions filled?	59.8	29.7	7.8	2.8
How often do you run out of medicine?	64.9	29.7	4.2	1.2
How often do you skip a dose of your medicine before you go to the doctor?	57.6	33.4	6.9	2.1
How often do you miss taking you medicine when you feel better?	52.1	32.9	11.9	3.1
How often do you miss taking your medicine when you feel sick?	73.4	22.6	4.0	0.0
How often do you miss taking your medicine when you are careless?	33.0	45.2	16.4	5.4
How often do you change the dose of your medicines to suit your needs (like when you take more or less pill than you’re supposed to)?	59.8	32.3	6.9	1.0
How often do you forget to take your medicine when you are supposed to take it more than once a day?	52.4	40.9	6.7	0.0
How often do you put off refilling your medicines because they cost too much money?	71.3	23.9	3.6	1.2
How often do you plan ahead and refill your medicines before they run out?	1.7	24.7	24.7	49.0
**Perfect adherence (summary score = 12)**				
Non-adherent	87.8			
Perfectly adherent	12.2			
**Level of adherence**				
Low adherence	61.3			
High adherence	38.7			
**Adherence to taking medications correctly subscale**, mean (SD)	12.54 (4.07)			
**Adherence to refilling medications on schedule subscale**, mean (SD)	6.09 (1.96)			
**ARMS summary score**, mean (SD)	18.63 (5.36)			

Note: Values represent n and %, unless stated otherwise. Abbreviation: n, number of patients; SD, standard deviation; ARMS, Adherence to Refills and Medications Scale

**Table 4:** ARMS questionnaire responses (%) in hyperlipidemia

Items	None of the time	Some of the time	Most of the time	All the time
How often do you forget to take your medicine?	40.6	55.5	3.9	0.0
How often do you decide not to take your medicine?	63.3	32.4	4.3	0.0
How often do you forget to get prescriptions filled?	49.7	47.9	1.5	0.9
How often do you run out of medicine?	50.5	41.5	8.0	0.0
How often do you skip a dose of your medicine before you go to the doctor?	55.9	40.2	3.9	0.0
How often do you miss taking you medicine when you feel better?	55.5	37.1	6.7	0.6
How often do you miss taking your medicine when you feel sick?	58.1	34.8	7.1	0.0
How often do you miss taking your medicine when you are careless?	36.5	53.6	9.9	0.0
How often do you change the dose of your medicines to suit your needs (like when you take more or less pill than you’re supposed to)?	59.2	39.5	1.3	0.0
How often do you forget to take your medicine when you are supposed to take it more than once a day?	55.9	39.5	4.5	0.0
How often do you put off refilling your medicines because they cost too much money?	62.6	24.4	6.7	6.3
How often do you plan ahead and refill your medicines before they run out?	1.9	44.1	26.6	27.4
**Perfect adherence (summary score = 12)**				
Non-adherent	89.0			
Perfectly adherent	11.0			
**Level of adherence**				
Low adherence	66.7			
High adherence	33.3			
**Adherence to taking medications correctly subscale**, mean (SD)	12.18 (3.63)			
**Adherence to refilling medications on schedule subscale**, mean (SD)	6.88 (2.05)			
**ARMS summary score**, mean (SD)	19.06 (5.15)			

Note: Values represent n and %, unless stated otherwise. Abbreviations: n, number of patients; SD, standard deviation; ARMS, Adherence to Refills and Medications Scale

The results of the univariate analysis are presented in Table 5. Female gender, higher age, low education, no health insurance or only private, obesity, exercising 1–2 days per week, low satisfaction with physician consultation, shorter consultation length, high perceived threat of illness, fair, bad, or very bad general health, and uncontrolled level of HbA1c were significantly (*p* < 0.05) associated with a lower level of the ARMS summary score, i.e., poorer medication adherence, in diabetic patients. In hypertension, medication adherence was significantly lower in patients who were males, not married, employed, low-educated, with no health insurance or only private, with higher BMI, smokers, not engaging in physical exercise, reporting lower physician consultation length, not being well-informed about the prescribed medication, with low satisfaction with physician consultations, negative NCD and high level of perceived threat related to their disease, fair, bad, or very bad general health, and uncontrolled blood pressure. Furthermore, other factors that achieved statistical significance, although the pattern was not as clear, were age, drinking, and the number of comorbidities. Finally, in hyperlipidemia, a higher ARMS summary score was significantly associated with lower education, no public health insurance, smoking, lower physical activity, shorter physician consultations, lower patient satisfaction with the consultation, higher level of concerns and weaker ideas about the necessity of taking the prescribed medicines, fair, bad, or very bad general health, shorter duration of taking the medication, uncontrolled LDL, and low HDL. Other significant factors were age, drinking, and the total number of medications.

**Table 5:** Univariate analysis of factors associated with the ARMS summary score

Factors	Diabetes		Hypertension		Hyperlipidemia	
	Mean (SD)	*p*-value	Mean (SD)	*p*-value	Mean (SD)	*p*-value
**Sex**		0.005		< 0.001		0.622
Male	15.03 (3.01)		19.58 (5.17)		19.00 (5.41)	
Female	15.89 (3.68)		17.37 (5.37)		19.16 (4.68)	
**Age**		0.038		< 0.001		< 0.001
≤ 49 years	15.63 (3.07)		18.62 (6.35)		18.84 (5.15)	
50–59 years	15.77 (4.22)		21.17 (5.92)		17.68 (5.33)	
60–69 years	15.14 (3.70)		18.44 (5.28)		21.07 (5.43)	
≥ 70 years	14.91 (3.96)		17.84 (4.78)		18.49 (4.77)	
**Married**		0.424		0.001		0.421
No	15.70 (3.56)		19.43 (5.40)		19.51 (5.73)	
Yes	15.48 (3.42)		18.23 (5.31)		18.84 (4.85)	
**Employed**		0.628		< 0.001		0.203
Yes	15.51 (3.45)		20.07 (5.94)		18.65 (5.15)	
No	15.67 (3.52)		17.76 (4.78)		19.31 (5.15)	
**Education level**		0.462		< 0.001		0.021
Low education	15.84 (3.83)		19.08 (5.50)		19.55 (5.32)	
High education	15.35 (3.13)		16.83 (4.37)		18.26 (4.77)	
**Health insurance status**		< 0.001		0.011		< 0.001
Public	15.54 (3.45)		18.35 (5.09)		19.11 (4.89)	
Public & private	14.47 (2.00)		19.28 (6.52)		18.03 (5.51)	
No health insurance or only private	17.33 (4.48)		21.19 (4.42)		23.09 (5.43)	
**BMI categories**		0.011		0.006		0.714
Underweight or normal weight	15.81		18.07		18.85	
Overweight	14.94		18.53		19.24	
Obese	15.96		20.22		19.08	
**Smoking**		0.366		< 0.001		< 0.001
No	15.61		17.15		17.78	
Yes	15.53		22.01		21.07	
**Drinking alcohol frequency**		0.780		< 0.001		< 0.001
2–4 times per month or less	15.55 (3.47)		18.25 (5.07)		18.62 (5.00)	
At least 2–3 times weekly	15.74 (3.57)		20.63 (6.35)		22.23 (5.23)	
**Exercise per week**		< 0.001		< 0.001		< 0.001
0 days	15.78 (4.47)		20.20 (5.54)		20.03 (5.44)	
1–2 days	16.21 (3.45)		17.56 (4.89)		18.73 (4.84)	
3 + days	15.00 (2.89)		16.29 (4.24)		17.92 (4.74)	
**Patient satisfaction with consultation**		< 0.001		< 0.001		< 0.001
Low satisfaction	18.29 (5.50)		22.61 (6.76)		21.57 (5.37)	
High satisfaction	15.30 (3.07)		18.20 (5.01)		18.30 (4.84)	
**Consultation length**		0.035		< 0.001		0.004
≤ 10 min	18.12 (5.39)		21.11 (4.49)		19.54 (5.04)	
11–20 min	15.73 (3.88)		18.15 (5.40)		19.94 (5.06)	
≥ 21 min	15.30 (2.94)		18.24 (5.39)		18.30 (5.17)	
**Self-perceived knowledge adequacy about the medication**		0.928		< 0.001		< 0.001
Yes	15.56 (3.45)		16.84 (4.97)		18.27 (4.88)	
No	15.62 (3.55)		21.70 (4.57)		20.45 (5.34)	
**BMQ NCD**		0.054		< 0.001		< 0.001
Negative cost-benefit	16.57 (3.95)		23.59 (5.08)		22.59 (4.86)	
Positive cost-benefit	15.52 (3.44)		17.19 (4.52)		17.95 (4.73)	
**BIPQ Level of illness threat**		< 0.001		< 0.001		0.246
Low threat	14.49 (2.52)		18.08 (5.60)		18.91 (5.23)	
High threat	15.94 (3.68)		19.36 (4.95)		19.50 (4.91)	
**Good or very good general health**		< 0.001		< 0.001		0.040
No	16.22 (3.61)		19.59 (5.19)		19.36 (4.92)	
Yes	15.21 (3.35)		17.58 (5.36)		18.55 (5.51)	
**Number of comorbidities**		0.112		< 0.001		0.206
0	15.92 (3.67)		16.31 (4.81)		19.80 (4.79)	
1	15.49 (3.31)		20.19 (4.42)		18.83 (5.21)	
≥ 2	15.28 (3.36)		18.85 (6.09)		18.88 (5.28)	
**Time taking medication for main disease**		0.824		0.298		< 0.001
0–4 years	16.06 (4.74)		19.77 (5.85)		19.96 (4.97)	
5–9 years	15.68 (3.23)		18.64 (5.28)		19.13 (5.35)	
10–19 years	15.52 (3.17)		18.31 (5.43)		19.22 (4.81)	
20 + years	15.47 (3.49)		18.39 (4.93)		16.54 (5.22)	
**Total number of medications (for all conditions)**		0.674		0.702		< 0.001
1–2	15.71 (3.58)		18.84 (6.06)		18.51 (4.99)	
3–4	15.52 (3.47)		18.64 (4.92)		21.23 (5.24)	
5+	15.42 (3.31)		18.31 (4.83)		17.12 (4.25)	
**Type of diabetes**		0.461				
Type1	15.33 (2.98)					
Type2	16.07 (4.25)					
**Type of antidiabetic medication**		0.841				
Only oral medication	15.99 (4.27)					
Insulin with or without oral medication	15.37 (2.98)					
**HbA1c**		0.014				
Less than 7%	15.21 (3.40)					
Greater than or equal to 7%	15.85 (3.52)					
**Blood pressure**				< 0.001		
Greater than 130/80 mm Hg			20.27 (5.68)			
Less than or equal to 130/80 mm Hg			16.69 (4.22)			
**LDL**						0.006
Greater than or equal to 100 mg/dL)					19.61 (5.29)	
Less than 100 mg/dL					18.17 (4.64)	
**HDL**						< 0.001
Less than or equal to 40 mg/dL for male or less than or equal to 50 mg/dL for female					21.31 (4.66)	
Greater than 40 mg/dL for male or greater than 50 mg/dL for female					17.84 (4.92)	
**Triglyceride**						0.487
Greater than or equal to 150 mg/dL					19.00 (5.38)	
Less than 150 mg/dL					19.27 (4.51)	

Abbreviations: SD, standard deviation; BIPQ, Brief Illness Perception Questionnaire; BMI, body mass index; BMQ NCD, Beliefs about Medicines Questionnaire necessity-concerns scales differential; HbA1C, hemoglobin A1C; HDL, high-density lipoprotein; LDL, low-density lipoprotein

According to the results of the multiple linear regression analysis, female gender, no health insurance or only private, high perceived threat of illness, and type 2 diabetes were significantly associated with higher ARMS summary score, i.e., lower medication adherence, in diabetic patients, whereas higher age (over 70 years group), having both public and private health insurance, higher BMI (although the effect declines in obese participants), high satisfaction with physician consultations, lengthier consultations, good or very good general health, and two or more comorbidities were independently improving medication adherence in this group (Table 6). In hypertension, no health insurance or only private, smoking, frequent drinking, exercising 1–2 days per week, self-perceived knowledge inadequacy about the medicines, and presence of comorbidities were independent predictors of lower medication adherence, while female gender, marriage, high education, being overweight, lengthier consultations, positive NCD, higher number of medications, and controlled blood pressure had a significant positive association with medication adherence. Finally, in hyperlipidemia, patients that were not employed, overweight, smokers, frequent drinkers, taking three to four medicines for all their conditions, had no health insurance or only private, and with low triglyceride levels were significantly less adherent, whereas medication adherence was significantly better in participants that were older, more often exercising, highly satisfied with the consultations, with fewer concerns and stronger ideas of need towards their medicines, controlled LDL, and high HDL.

**Table 6:** Multiple linear regression analysis

Factors	Diabetes^a^		Hypertension^b^		Hyperlipidemia^c^	
	Beta (95% CI)	*p*-value	Beta (95% CI)	*p*-value	Beta (95% CI)	*p*-value
**Sex** (Ref.: Male)						
Female	0.64 (0.01–1.28)	0.048	-0.82 (-1.45–0.19)	0.011	0.62 (-0.20-1.44)	0.139
**Age** (Ref.: ≤49 years)						
50–59 years	0.15 (-0.64-0.93)	0.717	1.37 (-0.12-2.86)	0.072	-2.42 (-3.74–1.11)	< 0.001
60–69 years	-0.92 (-2.33-0.50)	0.203	0.22 (-1.30-1.73)	0.778	-1.55 (-2.98–0.13)	0.032
≥ 70 years	-1.65 (-3.00–0.30)	0.017	-0.42 (-2.11-1.26)	0.623	-2.82 (-4.53–1.12)	0.001
**Married** (Ref.: No)						
Yes	-0.33 (-0.94-0.28)	0.291	-0.82 (-1.43–0.21)	0.008	-0.70 (-1.48-0.08)	0.078
**Employed** (Ref.: Yes)						
No	-0.21 (-0.90-0.48)	0.559	-0.19 (-1.14-0.77)	0.697	1.77 (0.41–3.12)	0.011
**Education** (Ref.: Low level)						
High level	-0.40 (-1.02-0.21)	0.198	-1.34 (-2.16–0.53)	0.001	-0.09 (-1.13-0.95)	0.867
**Health insurance status** (Ref.: Public)						
Public & private	-0.86 (-1.54–0.17)	0.014	-0.45 (-1.27-0.36)	0.277	-0.29 (-1.33-0.76)	0.592
No health insurance or only private	1.35 (0.19–2.51)	0.023	1.48 (0.18–2.78)	0.026	3.22 (1.54–4.90)	< 0.001
**BMI categories** (Ref.: Underweight or Normal weight)						
Overweight	-1.03 (-1.72–0.34)	0.004	-0.67 (-1.28–0.07)	0.030	1.09 (0.26–1.91)	0.010
Obese	-0.78 (-1.52–0.03)	0.043	-0.26 (-1.09-0.58)	0.548	0.62 (-0.34-1.57)	0.207
**Smoking** (Ref.: No)						
Yes	-0.47 (-1.08-0.14)	0.128	1.63 (0.87–2.40)	< 0.001	3.09 (2.36–3.81)	< 0.001
**Drinking alcohol frequency** (Ref.: Never)						
At least 2–3 times per week	0.43 (-0.28-1.13)	0.237	1.11 (0.01–2.21)	0.048	2.97 (1.68–4.27)	< 0.001
**Exercise per week** (Ref.: 0 days)						
1–2 days	0.25 (-0.69-1.19)	0.598	0.80 (0.17–1.44)	0.013	-1.17 (-2.04–0.30)	0.009
3 + days	-0.64 (-1.53-0.26)	0.163	0.24 (-0.74-1.22)	0.628	-2.21 (-3.04–1.38)	< 0.001
**Patient satisfaction with consultation** (Ref.: Low)						
High satisfaction	-2.05 (-3.45–0.65)	0.004	-1.12 (-2.35-0.11)	0.073	-2.41 (-3.54–1.29)	< 0.001
**Consultation length** (Ref.: ≤10 min)						
11–20 min	-1.92 (-3.72–0.12)	0.037	-1.25 (-2.17–0.34)	0.007	0.40 (-0.62-1.42)	0.440
≥ 21 min	-1.95 (-3.73–0.18)	0.031	-1.48 (-2.46–0.50)	0.003	0.35 (-0.65-1.34)	0.491
**Self-perceived knowledge adequacy about the medication** (Ref.: Yes)						
No	-0.27 (-0.91-0.37)	0.409	2.80 (2.23–3.37)	< 0.001	-0.37 (-1.24-0.51)	0.407
**BMQ NCD** (Ref.: Negative cost-benefit)						
Positive cost-benefit	-0.21 (-1.93-1.50)	0.807	-3.99 (-4.75–3.22)	< 0.001	-3.82 (-4.81–2.83)	< 0.001
**BIPQ Level of illness threat** (Ref.: Low threat)						
High threat	1.04 (0.44–1.64)	0.001	-0.54 (-1.15-0.06)	0.079	-0.52 (-1.48-0.44)	0.290
**Good or very good general health** (Ref.: No)						
Yes	-0.70 (-1.34–0.06)	0.032	-0.01 (-0.67-0.66)	0.988	-0.41 (-1.48-0.66)	0.450
**Number of comorbidities** (Ref.: None)						
1	-0.80 (-1.82-0.21)	0.121	3.01 (2.19–3.83)	< 0.001	0.09 (-1.04-1.23)	0.873
≥ 2	-1.49 (-2.61–0.37)	0.009	2.79 (1.75–3.83)	< 0.001	-0.96 (-2.39-0.48)	0.190
**Time taking medication for main disease** (Ref.: 0–4 years)						
5–9 years	0.15 (-1.20-1.50)	0.830	-0.76 (-1.74-0.21)	0.125	0.57 (-0.42-1.56)	0.259
10–19 years	-0.01 (-1.16-1.14)	0.986	0.03 (-0.96-1.02)	0.952	-0.14 (-1.17-0.89)	0.795
20 + years	0.61 (-0.55-1.77)	0.302	-0.71 (-1.78-0.37)	0.197	-0.71 (-1.97-0.55)	0.269
**Total number of medications for all conditions** (Ref.: 1–2)						
3–4	0.69 (-0.26-1.65)	0.155	-1.25 (-2.02–0.49)	0.001	2.70 (1.52–3.88)	< 0.001
5+	0.50 (-0.72-1.71)	0.423	-1.42 (-2.42–0.42)	0.006	0.16 (-1.28-1.61)	0.827
**Type of diabetes** (Ref.: Type 1)						
Type2	1.81 (0.99–2.63)	< 0.001				
**HbA1c** (Ref.: Less than 7%)						
Greater than or equal to 7%	0.30 (-0.31-0.90)	0.334				
**Blood pressure** (Ref.: Greater than 130/80 mm Hg)						
Less than or equal to 130/80 mm Hg			-2.47 (-3.05–1.89)	< 0.001		
**LDL** (Ref.: Greater than or equal to 100 mg/dL)						
Less than 100 mg/dL					-3.78 (-5.05–2.50)	< 0.001
**HDL** (Ref.: Less than or equal to 40 mg/dL for male or less than or equal to 50 mg/dL for female)						
Greater than 40 mg/dL for male or greater than 50 mg/dL for female					-3.05 (-4.00–2.10)	< 0.001
**Triglyceride** (Ref.: Greater than or equal to 150 mg/dL)						
Less than 150 mg/dL					3.20 (1.87–4.53)	< 0.001
**Constant**	19.67 (16.51–22.83)	< 0.001	23.94 (21.80-26.09)	< 0.001	25.62 (23.27–27.97)	< 0.001

Note: ^a^ Observations: 518, F(31, 486) = 4.91, *p* < 0.001, R^2^: 0.224. ^b^ Observations: 721, F(30, 690) = 42.82, *p* < 0.001, R^2^: 0.574. ^c^ Observations: 433, F(32, 412) = 28.96, *p* < 0.001, R^2^: 0.623. Abbreviations: BIPQ, Brief Illness Perception Questionnaire; BMI, body mass index; BMQ NCD, Beliefs about Medicines Questionnaire necessity-concerns scales differential; HbA1C, hemoglobin A1C; HDL, high-density lipoprotein; LDL, low-density lipoprotein

## Discussion

The primary aim of this research was to investigate the prevalence of medication non-adherence and its determinants in patients with diabetes, hypertension, and hyperlipidemia. Our study confirmed the presence of suboptimal medication adherence among people with diabetes, hypertension, and hyperlipidemia in Greece. Specifically, perfect medication adherence was reported by only 16%, 12%, and 11% for diabetes, hypertension, and hyperlipidemia, respectively. However, these estimates may underestimate the true magnitude of non-adherence in Greece. This is due to the fact that most participants were recruited during clinic visits. Many studies have demonstrated that medication compliance increases significantly during short time intervals immediately preceding and following clinic visits. This phenomenon is also known as white coat adherence and it may lead to misinterpretations of the overall adherence rate and the effectiveness of treatment [26–28]. For the purpose of comparison, we also used the median of the ARMS summary score for the total sample as a cut-off to differentiate between low and high adherence. Low adherence was again more frequent in hyperlipidemia (66.7%), followed closely by hypertension (61.3%), whereas the poor compliance rate was much lower in diabetes (38.8%). Interestingly, the most frequently reported non-adherence dimensions in all three conditions was timely prescription refill. Furthermore, the higher mean ARMS summary score in hyperlipidemic patients compared with hypertensive participants was mainly driven by the significantly (*p* < 0.05 in post-hoc analysis) larger mean refilling non-adherence subscale level in hyperlipidemia.

The above rate differences between patient groups can be explained by the fact that lipid- and blood-pressure-lowering medications are mainly used for primary prevention, In contrast, glucose-lowering medications have clearer morbidity and mortality implications which can be easily understood by patients [29]. Non-adherence is considerably more common in mainly asymptomatic chronic conditions, such as hyperlipidemia and hypertension, where treatment does not produce immediate and noticeable benefits for the patient [30–32]. Therefore, educating patients about the potential health effects of the condition and the health benefits of medication treatment could constitute a crucial intervention to promote medication adherence [32].

Comparison of levels of adherence between published studies is not always an easy task since reported non-adherence rates largely depend on the setting, the patients enrolled, and measurement methods [11]. Notably, information about adherence rates in chronic diseases in Greece has been to date somewhat scarce by comparison with the abundance of data for other countries. Regarding antihypertensives, good secondary adherence was associated with only 15% of subjects attending a Greek general public hospital [10]. In another survey, the complete medication adherence rate was 48% among patients with hypertension recruited from a center for hypertension in a Greek general public hospital [33]. In a multinational online survey, based on the Morisky questionnaire 50.2% of participants self-reported as being non-adherent to antihypertensive medicines, of whom 57.2% were intentionally non-adherent [34]. Furthermore, only 57.3% of patients with type 2 diabetes from various outpatient care centers throughout Greece were highly adherent to oral glucose-lowering treatment [35]. On the other hand, medication adherence was *determined* / shown to be much higher in another study among patients with diabetes attending public and private outpatient clinics, but only 14.1% of the subjects were diagnosed with type 1 diabetes [36]. Liatis et al. [37] reported that 7% of all subjects from a national type 2 diabetes registry had poor compliance with the prescribed medication. Furthermore, about 13–15% of patients with type 2 diabetes attending primary and secondary care practices in Greece reported missing at least one medication dose in the previous week [38]. Finally, a number of other studies have also been identified which investigated adherence in Greece: however, they focused on specific medications or modalities of treatment in clinical trial settings [39–42].

Our regression analysis showed that different factors affect non-adherence in opposite directions across the three chronic conditions under discussion. Older adults appear to be more adherent to their regimen compared with younger persons in hyperlipidemia and diabetes patient groups, but not in hypertension groups. The positive influence of older age corroborates the findings of several previous studies [43], while others showed no relationship between adherence and age or a negative effect [44, 45]. Although older age may be associated with various barriers to adherence, such as cognitive decline, multiple chronic conditions and multiple medications, their simpler and more ordered lifestyle may permit them to be more adherent [43]. Younger individuals are likely to have a busier schedule and a more recent diagnosis and, thus, less knowledge about the health implications of the disease and non-adherence, in addition to some degree of rebelliousness [46]. Therefore, adherence-improving interventions should consider younger age cohorts as well. Regarding the other demographic factors, female gender was related to lower adherence in diabetes and improved adherence in hypertension, while the effect did not reach statistical significance in hyperlipidemia. Moreover, being married increased medication adherence in hypertension, but the regression coefficients were not significant in the other groups. The literature shows that, in general, demographic factors tend to have an inconsistent impact on adherence [43]. However, it is hypothesized that spouses provide practical support and supervision, thus promoting more adherent behavior [47].

One crucial finding was that patients with no health insurance or only private were significantly less adherent in all conditions, while having both public and private health insurance was associated with higher adherence in diabetes. Health insurance coverage is an important factor of medication adherence as it is related to the presence of financial constraints confronted by the patient. This may be associated with the cost of medications, which imposes a great burden on many patients, as well as access to health services and prescribers [48]. Regarding patients’ socioeconomic background, highly educated hypertensive participants were found to be more adherent, while not being employed increased non-adherence in hyperlipidemia. However, overall, the impact of employment and education on medication adherence in chronic diseases has been characterized as uncertain [49]. The negative effect of not being employed may be related to a lower disposable income, which is a practical barrier to adherence [50]. A higher education level may also be associated with a higher income status through better job opportunities. Furthermore, health literacy and medication knowledge, especially as regards understanding about their side effects, is positively linked to patient educational level [51], while it contributes to better cooperation between health providers and higher educated patients [52]. On the other hand, better educated individuals are more likely to seek to be involved in decisions about their health. As a result, if they feel they are not involved enough, they might intentionally non-adhere to their medication treatment [20].

Smoking and more frequent alcohol intake in hypertension and hyperlipidemia were significant predictors of lower adherence. In contrast, the association of BMI and physical activity with adherence was not consistent across patient groups, different types of curvilinear relationships being observed. Specifically, patients with diabetes who were overweight and obese and those with hypertension who were overweight were significantly more adherent, whereas overweight hyperlipidemic participants were found to be less adherent. Furthermore, less regular physical activity in hyperlipidemia but exercising 1–2 days per week in hypertension tended to increase medication non-adherence. Effective management of diabetes, hypertension, and hyperlipidemia requires the modification of health behaviors, including smoking, drinking, dieting, and exercising. A positive correlation between medication adherence and healthy behaviors can be explained by the fact that self-efficacy (confidence in one’s own ability to achieve certain goals) is associated with both practices [53]. In general, people who engage in unhealthy behaviors can be considered to have less interest in investing in their health [54]. Therefore, interventions aiming at promoting healthy behaviors in the self-management of chronic conditions may also indirectly enhance medication adherence [54]. Furthermore, some detrimental habits are intrinsically related to social activities, such as drinking. Therefore, restriction of alcohol consumption due to taking medicines could lead to non-adherence [30].

Patients’ understanding, perceptions, and beliefs constitute powerful predictors of medication adherence. Medication adherence appears to be associated with the quality, duration, and frequency of doctor-patient interaction [55]. This explains the positive association of adherence with consultation length in diabetes and hypertension and with patient satisfaction with physician consultation in diabetes and hyperlipidemia that was established in our study. Adherence to antihypertensive medicines was identified as being higher in patients who felt better informed about their disease and their prescribed medication. A major barrier to medication adherence is patients’ poor understanding of important issues related to the disease and its treatment [55]. Increasing patients’ knowledge may enhance their skills in managing their condition, improve tolerability and efficacy of medications, and render them better partners with healthcare providers in reaching health goals [56]. However, patients’ perceptions about their knowledge adequacy does not necessarily correspond to how well-informed they actually are [33]. Effective doctor-patient communication is crucial for providing relevant information to the patient, acknowledging the patient’s needs and perspectives, co-establishing attainable therapy goals, and regulating each patient’s emotions and expectations [57, 58].

Notably, a positive necessity-concerns differential was significantly associated with a lower probability of low medication adherence in hypertension and hyperlipidemia. In other words, patients with stronger beliefs in the necessity of their treatment relative to concerns about its related side effects were more likely to be adherent. However, the Necessity-Concerns Framework is more predictive of intentional non-adherence than unintentional [20, 59, 60]. Unintentional non-adherence arises mainly from resource limitations (e.g., access problems) and personal constraints (e.g., forgetfulness) or from communication problems between patients and healthcare providers [61]. The Necessity-Concerns differential reflects individuals’ balance of beliefs which they consider while making their adherent-related decisions [20]. Interestingly, a more threatening view of illness was associated with lower adherence in patients with diabetes, in line with the common sense model [62]. More threatening perceptions of diabetes may lead to higher concerns about its treatment and its side effects [63]. The direction of the effect in diabetes may reflect the impact of fear of iatrogenic hypoglycemia, which drives patients to be less adherent to their prescribed antihyperglycemic medicines. The actual disease severity may differ from the lived experience of illness. Appropriate health messages should be developed to enhance medication adherence by helping patients to understand the health threats related to their disease and the potential benefits of compliance to treatment guidelines [64]. In general, comorbidities and patient history have an inconsistent effect on medication adherence [45].

Regarding the clinical factors, their impact differed across patient groups. Participants with type 2 diabetes were more likely to have low adherence compared with those with type 1 diabetes. This could reflect the effect of both the duration of diabetes and the type of therapy used. Furthermore, better general health was associated with a statistically significant higher adherence in diabetes. This result may have different explanations. A better self-perceived health status may be associated with better self-control and more positive ideas about the disease, thus enhancing medication adherence [65]. Furthermore, a good subjective health rating may be perceived by patients as a sign that the treatment is effective, which may motivate them to be more adherent. On the other hand, a bad health status may reflect the presence of comorbidities or greater disease severity. The presence of comorbidities was associated with higher non-adherence in hypertension. Nevertheless, patients of poorer health are usually more inclined to adhere to their treatment [64]. In our study, diabetic patients with two or more comorbidities were found to be more adherent compared with those without concurrent diseases. In addition, hyperlipidemic patients taking 3–4 medicines were significantly less adherent, while taking more medicines was positively correlated with adherence in hypertension. The latter finding is interesting as higher daily total medicine burden is considered a risk factor of medication non-adherence. This is because a larger number of medicines may overwhelm patients due to regimen complexity [66]. However, there are several studies that report the opposite or no effect [67–69], as it appears that non-adherence is more strongly correlated with the frequency of prescribed dosing than the number of medications [70].

Glucose levels were not associated with medication adherence. This may be attributed to the fact that glycemic control is influenced by multiple factors apart from medication adherence, such as type of diet and level of exercise, degree of insulin deficit, and effectiveness of the medications used [71]. Furthermore, uncontrolled blood pressure increased the probability of low adherence in hypertensive patients. The association between controlled blood pressure and high adherence is well-documented in the literature [10, 31, 72]. High LDL and low HDL and triglyceride levels were independently associated with higher non-adherence in hyperlipidemia. Although it is well established that medication adherence is related to control of LDL [73–75] and higher HDL [71, 75, 76], the level of triglycerides is mostly linked to adherence to the recommended diet [77]. Therefore, a positive association between medication adherence and triglyceride levels may be an indication that patients consider their medication adherence as a substitute for behavioral control [53, 78].

This study has possible limitations that should be considered. First, measurement of medication adherence (as well as other clinical parameters) was based on self-reports, which may have led to measurement imprecision. However, there is to date no gold standard measure of medication adherence, and each method has its own strengths and weaknesses and the simplest and one of the most frequently used adherence measure in the literature is patient self-reporting [61]. Second, survey data are generally susceptible to certain biases (i.e., report, recall). Medication adherence may had been exaggerated by several patients as data were collected via personal interviews and during routine clinical visits. Third, the survey was conducted during the pandemic [79], which may have influenced medication adherence as well as its association with its factors. Fourth, due to the cross-sectional design of the study, causality cannot be established between medication adherence and its determinants.

## Conclusions

In conclusion, non-adherence to pharmacological treatment was found to be quite common in diabetes, hypertension, and hyperlipidemia in Greece. Notably, its prevalence was higher in hypertension and hyperlipidemia, which are generally asymptomatic conditions and the benefits of the treatment are not immediately noticeable by patients. Medication adherence constitutes a complex behavioral process, and the key reasons for non-compliance were found to be different among the patient groups. Whether adherence-enhancing interventions are worthwhile can only be determined after careful consideration by the patient of both the costs and benefits of adherence [80]. Nevertheless, some factors emerged as being particularly important and more consistent in our analysis. In this respect, strategies to improve adherence to pharmacotherapy should focus on the health education of younger people, patients with uncontrolled disease, and individuals engaging in unhealthy behaviors. Furthermore, far greater attention should also be given to structural barriers, this being illustrated by the consistent negative impact of the lack of public health insurance on medication adherence. More importantly, patient-centered interventions and measures to improve doctor-patient communication and to increase patient satisfaction are needed to establish a partnership between health professionals and patients. In addition, more effort should be directed towards changing patients’ negative perceptions about their treatment and improving their knowledge about their illness and their prescribed medications. Future research should distinguish between the intentional and unintentional aspects of medication non-adherence, while a better understanding of the factors that influence not only each chronic condition differently, but also each stage of the process, i.e., initiation, implementation, and discontinuation, is required to provide a more solid basis for the designing of adherence-improving strategies.

## References

[1] Megari K (2013) Quality of life in Chronic Disease patients. Health Psychol Res 1(3):e27. 10.4081/hpr.2013.e27 26973912 10.4081/hpr.2013.e27 PMC4768563

[2] Van Wilder L, Clays E, Devleesschauwer B, Pype P, Boeckxstaens P, Schrans D, De Smedt D (2020) Health-related quality of life in patients with non-communicable disease: study protocol of a cross-sectional survey. BMJ Open 10(9):e037131. 10.1136/bmjopen-2020-037131 32912984 10.1136/bmjopen-2020-037131 PMC7485234

[3] Ge L, Ong R, Yap CW, Heng BH (2019) Effects of chronic diseases on health-related quality of life and self-rated health among three adult age groups. Nurs Health Sci 21(2):214–222. 10.1111/nhs.12585 30537214 10.1111/nhs.12585

[4] Walsh CA, Cahir C, Tecklenborg S, Byrne C, Culbertson MA, Bennett KE (2019) The association between medication non-adherence and adverse health outcomes in ageing populations: a systematic review and meta-analysis. Br J Clin Pharmacol 85(11):2464–2478. 10.1111/bcp.14075 31486099 10.1111/bcp.14075 PMC6848955

[5] World Health Organization (2003) Adherence to long-term therapies: evidence for action. World Health Organization, Geneva

[6] Zullig LL, Gellad WF, Moaddeb J, Crowley MJ, Shrank W, Granger BB, Granger CB, Trygstad T, Liu LZ, Bosworth HB (2015) Improving diabetes medication adherence: successful, scalable interventions. Patient Prefer Adherence 9:139–149. 10.2147/PPA.S69651 25670885 10.2147/PPA.S69651 PMC4315534

[7] Huther J, von Wolff A, Stange D, Harter M, Baehr M, Dartsch DC, Kriston L (2013) Incomplete medication adherence of chronically ill patients in German primary care. Patient Prefer Adherence 7:237–244. 10.2147/PPA.S38373 23569363 10.2147/PPA.S38373 PMC3615847

[8] Kontochristopoulos G, Kouris A, Chantzaras A, Petridis A, Yfantopoulos J (2016) Improvement of health-related quality of life and adherence to treatment with calcipotriol-betamethasone dipropionate gel in patients with psoriasis vulgaris. Bras Dermatol 91(2):160–166. 10.1590/abd1806-4841.20164476 10.1590/abd1806-4841.20164476 PMC4861562 27192514

[9] Chantzaras A, Yfantopoulos J (2022) Association between medication adherence and health-related quality of life of patients with diabetes. Hormones 21(4):691–705. 10.1007/s42000-022-00400-y 36219341 10.1007/s42000-022-00400-y PMC9552716

[10] Yiannakopoulou E, Papadopulos JS, Cokkinos DV, Mountokalakis TD (2005) Adherence to antihypertensive treatment: a critical factor for blood pressure control. Eur J Cardiovasc Prev Rehabil 12(3):243–249. 10.1097/00149831-200506000-00010 15942423 10.1097/00149831-200506000-00010

[11] Ingersgaard MV, Helms Andersen T, Norgaard O, Grabowski D, Olesen K (2020) Reasons for nonadherence to statins - a systematic review of reviews. Patient Prefer Adherence 14:675–691. 10.2147/PPA.S245365 32308373 10.2147/PPA.S245365 PMC7135196

[12] Cheen MHH, Tan YZ, Oh LF, Wee HL, Thumboo J (2019) Prevalence of and factors associated with primary medication non-adherence in chronic disease: a systematic review and meta-analysis. Int J Clin Pract 73(6):e13350. 10.1111/ijcp.13350 30941854 10.1111/ijcp.13350

[13] Kripalani S, Risser J, Gatti ME, Jacobson TA (2009) Development and evaluation of the adherence to refills and medications Scale (ARMS) among low-literacy patients with chronic disease. Value Health 12(1):118–123. 10.1111/j.1524-4733.2008.00400.x 19911444 10.1111/j.1524-4733.2008.00400.x

[14] McNaughton CD, Brown NJ, Rothman RL, Liu D, Kabagambe EK, Levy PD, Self WH, Storrow AB, Collins SP, Roumie CL (2017) Systolic blood pressure and biochemical Assessment of Adherence: a cross-sectional analysis in the Emergency Department. Hypertension 70(2):307–314. 10.1161/HYPERTENSIONAHA.117.09659 28652467 10.1161/HYPERTENSIONAHA.117.09659 PMC5531074

[15] Jankowska-Polanska B, Karniej P, Polanski J, Sen M, Swiatoniowska-Lonc N, Grochans E (2020) Diabetes Mellitus Versus Hypertension-Does Disease affect pharmacological adherence? Front Pharmacol 11:1157. 10.3389/fphar.2020.01157 32848766 10.3389/fphar.2020.01157 PMC7432322

[16] Parra DI, Romero Guevara SL, Rojas LZ (2019) Influential factors in adherence to the Therapeutic Regime in Hypertension and Diabetes. Invest Educ Enferm 37(3). 10.17533/udea.iee.v37n3e02 10.17533/udea.iee.v37n3e02 PMC7871498 31830400

[17] Horne R, Weinman J, Hankins M (1999) The beliefs about medicines questionnaire: the development and evaluation of a new method for assessing the cognitive representation of medication. Psychol Health 14(1):1–24. 10.1080/08870449908407311

[18] Komninis ID, Micheli K, Roumeliotaki T, Horne R (2013) Adaptation and validation of the beliefs about Medicines Questionnaire (BMQ) in primary care patients in Greece. Eur J Person Centered Healthc 1(1):224–231. 10.5750/ejpch.v1i1.655

[19] Cheiloudaki E, Alexopoulos EC (2019) Adherence to treatment in Stroke patients. Int J Environ Res Public Health 16(2):196. 10.3390/ijerph16020196 30641978 10.3390/ijerph16020196 PMC6351941

[20] Foot H, La Caze A, Gujral G, Cottrell N (2016) The necessity-concerns framework predicts adherence to medication in multiple illness conditions: a meta-analysis. Patient Educ Couns 99(5):706–717. 10.1016/j.pec.2015.11.004 26613666 10.1016/j.pec.2015.11.004

[21] Moss-Morris R, Weinman J, Petrie K, Horne R, Cameron L, Buick D (2002) The revised illness perception questionnaire (IPQ-R). Psychol Health 17(1):1–16. 10.1080/08870440290001494

[22] Broadbent E, Petrie KJ, Main J, Weinman J (2006) The brief illness perception questionnaire. J Psychosom Res 60(6):631–637. 10.1016/j.jpsychores.2005.10.020 16731240 10.1016/j.jpsychores.2005.10.020

[23] Karademas EC, Bakouli A, Bastounis A, Kallergi F, Tamtami P, Theofilou M (2008) Illness perceptions, illness-related problems, subjective health and the role of perceived primal threat: preliminary findings. J Health Psychol 13(8):1021–1029. 10.1177/1359105308097967 18987075 10.1177/1359105308097967

[24] Siarava E, Markoula S, Pelidou SH, Kyritsis AP, Hyphantis T (2020) Psychological distress symptoms and illness perception in patients with epilepsy in Northwest Greece. Epilepsy Behav 102:106647. 10.1016/j.yebeh.2019.106647 31785484 10.1016/j.yebeh.2019.106647

[25] Kotsis K, Voulgari PV, Tsifetaki N, Drosos AA, Carvalho AF, Hyphantis T (2014) Illness perceptions and psychological distress associated with physical health-related quality of life in primary Sjogren’s syndrome compared to systemic lupus erythematosus and rheumatoid arthritis. Rheumatol Int 34(12):1671–1681. 10.1007/s00296-014-3008-0 24769916 10.1007/s00296-014-3008-0

[26] Burnier M, Santschi V, Favrat B, Brunner HR (2003) Monitoring compliance in resistant hypertension: an important step in patient management. J Hypertens Suppl 21(2):S37–42. 10.1097/00004872-200305002-00007 12929906 10.1097/00004872-200305002-00007

[27] Modi AC, Ingerski LM, Rausch JR, Glauser TA, Drotar D (2012) White coat adherence over the first year of therapy in pediatric epilepsy. J Pediatr 161(4):695–699e691. 10.1016/j.jpeds.2012.03.059 22608905 10.1016/j.jpeds.2012.03.059 PMC3426618

[28] Zueger T, Gloor M, Lehmann V, Melmer A, Kraus M, Feuerriegel S, Laimer M, Stettler C (2020) White coat adherence effect on glucose control in adult individuals with diabetes. Diabetes Res Clin Pract 168:108392. 10.1016/j.diabres.2020.108392 32858099 10.1016/j.diabres.2020.108392

[29] Lemstra M, Nwankwo C, Bird Y, Moraros J (2018) Primary nonadherence to chronic disease medications: a meta-analysis. Patient Prefer Adherence 12:721–731. 10.2147/PPA.S161151 29765208 10.2147/PPA.S161151 PMC5944464

[30] Ogedegbe G, Harrison M, Robbins L, Mancuso CA, Allegrante JP (2004) Barriers and facilitators of medication adherence in hypertensive African americans: a qualitative study. Ethn Dis 14(1):3–12. 10.1177/23743735241241176 15002917

[31] Krousel-Wood MA, Muntner P, Islam T, Morisky DE, Webber LS (2009) Barriers to and determinants of medication adherence in hypertension management: perspective of the cohort study of medication adherence among older adults. Med Clin North Am 93(3):753–769. 10.1016/j.mcna.2009.02.007 19427503 10.1016/j.mcna.2009.02.007 PMC2702217

[32] Bosworth HB, Ngouyombo B, Liska J, Zullig LL, Atlani C, Beal AC (2018) The importance of cholesterol medication adherence: the need for behavioral change intervention programs. Patient Prefer Adherence 12:341–348. 10.2147/PPA.S153766 29563777 10.2147/PPA.S153766 PMC5846762

[33] Stavropoulou C (2012) Perceived information needs and non-adherence: evidence from Greek patients with hypertension. Health Expect 15(2):187–196. 10.1111/j.1369-7625.2011.00679.x 21496190 10.1111/j.1369-7625.2011.00679.x PMC5060616

[34] Morrison VL, Holmes EA, Parveen S, Plumpton CO, Clyne W, De Geest S, Dobbels F, Vrijens B, Kardas P, Hughes DA (2015) Predictors of self-reported adherence to antihypertensive medicines: a multinational, cross-sectional survey. Value Health 18(2):206–216. 10.1016/j.jval.2014.12.013 25773556 10.1016/j.jval.2014.12.013

[35] Doupis J, Alexandrides T, Elisaf M, Melidonis A, Bousboulas S, Thanopoulou A, Pagkalos EM, Avramidis I, Pappas A, Arvaniti E, Karamousouli E, Voss B, Tentolouris N (2019) Influence of supervised Disease understanding and diabetes self-management on adherence to oral glucose-lowering treatment in patients with type 2 diabetes. Diabetes Therapy 10:1407–1422. 10.1007/s13300-019-0648-9 31222592 10.1007/s13300-019-0648-9 PMC6612340

[36] Geitona M, Latsou D, Toska A, Saridi M (2018) Polypharmacy and Adherence among Diabetic patients in Greece. Consult Pharm 33(10):562–571. 10.4140/TCP.n.2018.562 30322433 10.4140/TCP.n.2018.562.

[37] Liatis S, Iraklianou S, Kazakos K, Mastorakos G, Milios K, Mouslech Z, Noutsou M, Pagkalos E, Sampanis C, Investigators A (2019) A Greek registry of current type 2 diabetes management, aiming to determine core clinical approaches, patterns and strategies. BMC Endocr Disord 19(1):39. 10.1186/s12902-019-0364-5 31023374 10.1186/s12902-019-0364-5 PMC6482543

[38] Karagiannis T, Liakos A, Branda ME, Athanasiadou E, Mainou M, Boura P, Goulis DG, LeBlanc A, Montori VM, Tsapas A (2016) Use of the diabetes medication choice decision aid in patients with type 2 diabetes in Greece: a cluster randomised trial. BMJ Open 6(11):e012185. 10.1136/bmjopen-2016-012185 28186933 10.1136/bmjopen-2016-012185 PMC5129072

[39] Liakos CI, Papadopoulos DP, Kotsis VT (2017) Adherence to treatment, Safety, Tolerance, and effectiveness of Perindopril/Amlodipine fixed-dose combination in Greek patients with hypertension and stable coronary artery disease: a pan-hellenic prospective observational study of Daily Clinical Practice. Am J Cardiovasc Drugs 17(5):391–398. 10.1007/s40256-017-0232-5 28466368 10.1007/s40256-017-0232-5

[40] Papazafiropoulou AK, Sotiropoulos A, Souliotis K, Parcharidis G, Deligiannis G, Gionakis G, Papoulis S, Skliros E (2013) Compliance and fixed-dose combination therapy in a sample of Greek hypertensive patients. Hellenic J Cardiol 54(3):230–232 23685663

[41] Tsioufis K, Douma S, Kallistratos MS, Manolis AJ (2019) Effectiveness and adherence to treatment with Perindopril/Indapamide/Amlodipine Single-Pill Combination in a Greek Population with Hypertension. Clin Drug Investig 39(4):385–393. 10.1007/s40261-019-00761-0 30790132 10.1007/s40261-019-00761-0

[42] Pagkalos E, Thanopoulou A, Sampanis C, Bousboulas S, Melidonis A, Tentolouris N, Alexandrides T, Migdalis I, Karamousouli E, Papanas N (2018) The real-life effectiveness and care patterns of type 2 diabetes management in Greece. Exp Clin Endocrinol Diabetes 126(1):53–60. 10.1055/s-0043-109242 28704857 10.1055/s-0043-109242

[43] Assawasuwannakit P, Braund R, Duffull SB (2015) A model-based meta-analysis of the influence of factors that impact adherence to medications. J Clin Pharm Ther 40(1):24–31. 10.1111/jcpt.12219 25328015 10.1111/jcpt.12219

[44] DiMatteo MR (2004) Variations in patients’ adherence to medical recommendations: a quantitative review of 50 years of research. Med Care 42(3):200–209. 10.1097/01.mlr.0000114908.90348.f9 15076819 10.1097/01.mlr.0000114908.90348.f9

[45] Kardas P, Lewek P, Matyjaszczyk M (2013) Determinants of patient adherence: a review of systematic reviews. Front Pharmacol 4:91. 10.3389/fphar.2013.00091 23898295 10.3389/fphar.2013.00091 PMC3722478

[46] Bruna-Barranco I, Lue A, Gargallo-Puyuelo CJ, Arroyo MT, Alfambra E, Montero J, Gomollon F (2019) Young age and tobacco use are predictors of lower medication adherence in inflammatory bowel disease. Eur J Gastroenterol Hepatol 31(8):948–953. 10.1097/MEG.0000000000001436 31107739 10.1097/MEG.0000000000001436

[47] Trivedi RB, Ayotte B, Edelman D, Bosworth HB (2008) The association of emotional well-being and marital status with treatment adherence among patients with hypertension. J Behav Med 31(6):489–497. 10.1007/s10865-008-9173-4 18780175 10.1007/s10865-008-9173-4 PMC3746832

[48] Ong SE, Koh JJK, Toh SES, Chia KS, Balabanova D, McKee M, Perel P, Legido-Quigley H (2018) Assessing the influence of health systems on type 2 diabetes Mellitus awareness, treatment, adherence, and control: a systematic review. PLoS ONE 13(3):e0195086. 10.1371/journal.pone.0195086 29596495 10.1371/journal.pone.0195086 PMC5875848

[49] Gast A, Mathes T (2019) Medication adherence influencing factors-an (updated) overview of systematic reviews. Syst Rev 8(1):112. 10.1186/s13643-019-1014-8 31077247 10.1186/s13643-019-1014-8 PMC6511120

[50] Hope HF, Binkley GM, Fenton S, Kitas GD, Verstappen SMM, Symmons DPM (2019) Systematic review of the predictors of statin adherence for the primary prevention of cardiovascular disease. PLoS ONE 14(1):e0201196. 10.1371/journal.pone.0201196 30653535 10.1371/journal.pone.0201196 PMC6336256

[51] Alkatheri AM, Albekairy AM (2013) Does the patients’ educational level and previous counseling affect their medication knowledge? Ann Thorac Med 8(2):105–108. 10.4103/1817-1737.109823 23741273 10.4103/1817-1737.109823 PMC3667438

[52] Uchmanowicz B, Chudiak A, Mazur G (2018) The influence of quality of life on the level of adherence to therapeutic recommendations among elderly hypertensive patients. Patient Prefer Adherence 12:2593–2603. 10.2147/PPA.S182172 30584283 10.2147/PPA.S182172 PMC6287422

[53] Han E, Sohn HS, Lee JY, Jang S (2017) Health behaviors and Medication Adherence in Elderly patients. Am J Health Promot 31(4):278–286. 10.4278/ajhp.150205-QUAN-709 26730557 10.4278/ajhp.150205-QUAN-709

[54] Yaguchi Y, Fujihara K, Yamada MH, Matsubayashi Y, Kitazawa M, Osawa T, Yamamoto M, Kaneko M, Yamanaka N, Seida H, Kodama S, Sone H (2020) Skipping breakfast, late-night eating and current smoking are associated with medication adherence in Japanese patients with diabetes. Prim Care Diabetes 14(6):753–759. 10.1016/j.pcd.2020.05.002 32527662 10.1016/j.pcd.2020.05.002

[55] Vermeire E, Hearnshaw H, Van Royen P, Denekens J (2001) Patient adherence to treatment: three decades of research. A comprehensive review. J Clin Pharm Ther 26(5):331–342. 10.1046/j.1365-2710.2001.00363.x 11679023 10.1046/j.1365-2710.2001.00363.x

[56] Conn VS, Ruppar TM, Enriquez M, Cooper PS (2016) Patient-centered outcomes of Medication Adherence interventions: systematic review and Meta-analysis. Value Health 19(2):277–285. 10.1016/j.jval.2015.12.001 27021763 10.1016/j.jval.2015.12.001 PMC4812829

[57] Yfantopoulos J, Protopapa M, Mantalias K, Chantzaras A, Koutsogianni K, Yfantopoulos P, Vassilopoulos D (2020) Patients’ and doctors’ beliefs about treatment and long-term adherence in Rheumatic diseases. Mediterr J Rheumatol 31(Suppl 1):152–162. 10.31138/mjr.31.1.152 32676574 10.31138/mjr.31.1.152 PMC7361187

[58] Yfantopoulos J, Protopapa M, Chantzaras A, Yfantopoulos P (2021) Doctors’ views and strategies to improve patients’ adherence to medication. Hormones 20(3):603–611. 10.1007/s42000-021-00294-2 33914291 10.1007/s42000-021-00294-2 PMC8082220

[59] Wroe A, Thomas M (2003) Intentional and unintentional nonadherence in patients prescribed HAART treatment regimens. Psychol Health Med 8(4):453–463. 10.1080/1354850310001604595 21974735 10.1080/1354850310001604595

[60] Clifford S, Barber N, Horne R (2008) Understanding different beliefs held by adherers, unintentional nonadherers, and intentional nonadherers: application of the necessity-concerns Framework. J Psychosom Res 64(1):41–46. 10.1016/j.jpsychores.2007.05.004 18157998 10.1016/j.jpsychores.2007.05.004

[61] Jimmy B, Jose J (2011) Patient medication adherence: measures in daily practice. Oman Med J 26(3):155–159. 10.5001/omj.2011.38 22043406 10.5001/omj.2011.38 PMC3191684

[62] Leventhal H, Phillips LA, Burns E (2016) The common-sense model of self-regulation (CSM): a dynamic framework for understanding illness self-management. J Behav Med 39(6):935–946. 10.1007/s10865-016-9782-2 27515801 10.1007/s10865-016-9782-2

[63] Fall E, Chakroun-Baggioni N, Bohme P, Maqdasy S, Izaute M, Tauveron I (2021) Common sense model of self-regulation for understanding adherence and quality of life in type 2 diabetes with structural equation modeling. Patient Educ Couns 104(1):171–178. 10.1016/j.pec.2020.06.023 32631647 10.1016/j.pec.2020.06.023

[64] DiMatteo MR, Haskard KB, Williams SL (2007) Health beliefs, disease severity, and patient adherence: a meta-analysis. Med Care 45(6):521–528. 10.1097/MLR.0b013e318032937e 17515779 10.1097/MLR.0b013e318032937e

[65] Kang CD, Tsang PP, Li WT, Wang HH, Liu KQ, Griffiths SM, Wong MC (2015) Determinants of medication adherence and blood pressure control among hypertensive patients in Hong Kong: a cross-sectional study. Int J Cardiol 182(C):250–257. 10.1016/j.ijcard.2014.12.064 25585359 10.1016/j.ijcard.2014.12.064

[66] Park HY, Seo SA, Yoo H, Lee K (2018) Medication adherence and beliefs about medication in elderly patients living alone with chronic diseases. Patient Prefer Adherence 12:175–181. 10.2147/PPA.S151263 29416319 10.2147/PPA.S151263 PMC5790098

[67] Rolnick SJ, Pawloski PA, Hedblom BD, Asche SE, Bruzek RJ (2013) Patient characteristics associated with medication adherence. Clin Med Res 11(2):54–65. 10.3121/cmr.2013.1113 23580788 10.3121/cmr.2013.1113 PMC3692389

[68] Kirkman MS, Rowan-Martin MT, Levin R, Fonseca VA, Schmittdiel JA, Herman WH, Aubert RE (2015) Determinants of adherence to diabetes medications: findings from a large pharmacy claims database. Diabetes Care 38(4):604–609. 10.2337/dc14-2098 25573883 10.2337/dc14-2098 PMC4370331

[69] Iihara N, Tsukamoto T, Morita S, Miyoshi C, Takabatake K, Kurosaki Y (2004) Beliefs of chronically ill Japanese patients that lead to intentional non-adherence to medication. J Clin Pharm Ther 29(5):417–424. 10.1111/j.1365-2710.2004.00580.x 15482384 10.1111/j.1365-2710.2004.00580.x

[70] Jin J, Sklar GE, Min Sen Oh V, Chuen Li S (2008) Factors affecting therapeutic compliance: a review from the patient’s perspective. Ther Clin Risk Manag 4(1):269–286. 10.2147/tcrm.s1458 18728716 10.2147/tcrm.s1458 PMC2503662

[71] Marinho FS, Moram CBM, Rodrigues PC, Leite NC, Salles GF, Cardoso CRL (2018) Treatment Adherence and Its Associated Factors in Patients with Type 2 Diabetes: Results from the Rio de Janeiro Type 2 Diabetes Cohort Study. J Diabetes Res 2018: 8970196. 10.1155/2018/8970196 10.1155/2018/8970196 PMC6288575 30599003

[72] Morisky DE, Ang A, Krousel-Wood M, Ward HJ (2008) Predictive validity of a medication adherence measure in an outpatient setting. J Clin Hypertens (Greenwich) 10(5):348–354. 10.1111/j.1751-7176.2008.07572.x 18453793 10.1111/j.1751-7176.2008.07572.x PMC2562622

[73] Katzmann JL, Mahfoud F, Bohm M, Schulz M, Laufs U (2019) Association of medication adherence and depression with the control of low-density lipoprotein cholesterol and blood pressure in patients at high cardiovascular risk. Patient Prefer Adherence 13:9–19. 10.2147/PPA.S182765 30587940 10.2147/PPA.S182765 PMC6302826

[74] Raebel MA, Dyer W, Nichols GA, Goodrich GK, Schmittdiel JA (2017) Relationships between Medication Adherence and Cardiovascular Disease risk factor control in Elderly patients with diabetes. Pharmacotherapy 37(10):1204–1214. 10.1002/phar.1994 28752555 10.1002/phar.1994 PMC5647232

[75] Mansur AP, Mattar AP, Tsubo CE, Simao DT, Yoshi FR, Daci K (2001) Prescription and adherence to statins of patients with coronary artery disease and hypercholesterolemia. Arq Bras Cardiol 76(2):111–118. 10.1590/s0066-782x2001000200002 11270314 10.1590/s0066-782x2001000200002

[76] Ladova K, Matoulkova P, Zadak Z, Macek K, Vyroubal P, Vlcek J, Morisky DE (2014) Self-reported adherence by MARS-CZ reflects LDL cholesterol goal achievement among statin users: validation study in the Czech Republic. J Eval Clin Pract 20(5):671–677. 10.1111/jep.12201 24917035 10.1111/jep.12201

[77] Amado L, Ferreira N, Miranda V, Meireles P, Povera V, Ferreira R, Fazendeiro-Matos J, Teixeira L, Paul C, Santos-Silva A, Costa E (2015) Self-reported medication adherence in patients with end-stage kidney Disease Undergoing Online-Haemodiafiltration. J Ren Care 41(4):231–238. 10.1111/jorc.12127 26768798 10.1111/jorc.12127

[78] Harrison TN, Derose SF, Cheetham TC, Chiu V, Vansomphone SS, Green K, Tunceli K, Scott RD, Marrett E, Reynolds K (2013) Primary nonadherence to statin therapy: patients’ perceptions. Am J Manag Care 19(4):e133–139 23725451

[79] Chantzaras A, Yfantopoulos J (2022) The impact of COVID-19 pandemic and its associations with government responses in Europe. Region Periphery 13(13):23–40. 10.12681/rp.30758

[80] Cleemput I, Kesteloot K, DeGeest S (2002) A review of the literature on the economics of noncompliance. Room for methodological improvement. Health Policy 59(1):65–94. 10.1016/s0168-8510(01)00178-6 11786175 10.1016/s0168-8510(01)00178-6

